# Evaluation of phase-locking to parameterized speech envelopes

**DOI:** 10.3389/fneur.2022.852030

**Published:** 2022-08-03

**Authors:** Wouter David, Robin Gransier, Jan Wouters

**Affiliations:** ExpORL, Department of Neurosciences, KU Leuven, Leuven, Belgium

**Keywords:** temporal processing, envelope modulations, envelope encoding, auditory steady-state responses (ASSR), speech processing

## Abstract

Humans rely on the temporal processing ability of the auditory system to perceive speech during everyday communication. The temporal envelope of speech is essential for speech perception, particularly envelope modulations below 20 Hz. In the literature, the neural representation of this speech envelope is usually investigated by recording neural phase-locked responses to speech stimuli. However, these phase-locked responses are not only associated with envelope modulation processing, but also with processing of linguistic information at a higher-order level when speech is comprehended. It is thus difficult to disentangle the responses into components from the acoustic envelope itself and the linguistic structures in speech (such as words, phrases and sentences). Another way to investigate neural modulation processing is to use sinusoidal amplitude-modulated stimuli at different modulation frequencies to obtain the temporal modulation transfer function. However, these transfer functions are considerably variable across modulation frequencies and individual listeners. To tackle the issues of both speech and sinusoidal amplitude-modulated stimuli, the recently introduced Temporal Speech Envelope Tracking (TEMPEST) framework proposed the use of stimuli with a distribution of envelope modulations. The framework aims to assess the brain's capability to process temporal envelopes in different frequency bands using stimuli with speech-like envelope modulations. In this study, we provide a proof-of-concept of the framework using stimuli with modulation frequency bands around the syllable and phoneme rate in natural speech. We evaluated whether the evoked phase-locked neural activity correlates with the speech-weighted modulation transfer function measured using sinusoidal amplitude-modulated stimuli in normal-hearing listeners. Since many studies on modulation processing employ different metrics and comparing their results is difficult, we included different power- and phase-based metrics and investigate how these metrics relate to each other. Results reveal a strong correspondence across listeners between the neural activity evoked by the speech-like stimuli and the activity evoked by the sinusoidal amplitude-modulated stimuli. Furthermore, strong correspondence was also apparent between each metric, facilitating comparisons between studies using different metrics. These findings indicate the potential of the TEMPEST framework to efficiently assess the neural capability to process temporal envelope modulations within a frequency band that is important for speech perception.

## Introduction

Natural speech is a complex and dynamic signal. One prominent component of the speech signal is the temporal envelope. The speech envelope contains slow modulations that are related to linguistic information at different timescales such as phrases, words, syllables, and phonemes (1, 2). The modulation spectrum of the speech envelope exhibits a prominent peak for slow modulations of 4–5 Hz (3, 4), which corresponds to the syllable rate in speech (1, 5–7). Since the timescales of these slow modulations coincide with spoken syllables, access to these envelope modulations and their representation in the neural signal traveling through the auditory pathway is essential for speech perception, especially when access to spectral information is limited (8–12).

Two main electrophysiological paradigms are often used to investigate the neural representation of these slow envelope modulations throughout the auditory pathway. One paradigm involves neural entrainment to speech, which refers to cortical responses that consistently phase-lock to slow modulations of the speech envelope (13). The relation between neural responses and the speech envelope through phase-locking has been established with magneto- and electroencephalography (MEG/EEG) (14–16). While listening to speech, the phase pattern of the neural response is consistent with the speech envelope modulations of 4–8 Hz (17, 18). Interestingly, several studies suggested that speech perception performance is associated with the degree of phase-locking to the speech envelope (19–21). In other words, neural phase-locked patterns that are less consistent with the speech envelope are associated with degraded speech perception. For example, higher disruption of neural phase-locking during listening with electrical transcranial stimulation has been shown to result in more degraded speech perception (22). These findings suggest that phase-locking to the speech envelope in the auditory pathway plays an important role in speech perception. Moreover, hierarchical linguistic structures – such as words, phrases, and sentences – are differentiated by input acoustical cues and linguistic higher-order comprehension processes (23–25). The phase-locked responses to speech from the auditory pathway consist of cortical activity at different timescales (or modulation frequency bands) that concurrently track different linguistic structures at different hierarchical levels.

Analyses of phase-locked responses to speech have pointed to distinct functional roles of the delta (1–4 Hz) and theta (4–8 Hz) bands. On the one hand, phase-locking in the delta band is largely associated with the amount of linguistic information in the speech signal (26, 27) and with the listener's proficiency in the language (28–30). By manipulating the different levels of linguistic structure in the speech signals, this can be studied. When listening to a stream of synthesized Chinese sentences, in which the sentence rate was not present in the envelope but was encoded in the linguistic structure, native Chinese listeners did show phase-locking at the sentence rate while native English listeners did not (29). Neural phase-locking is also associated with lexical, syntactic, and/or semantic changes in the linguistic content when the speech is comprehended. The theta band (4–8 Hz), on the other hand, seems to be more dependent on the saliency of the perceived acoustic envelope. To assess how envelope modulations at these low frequencies are processed by the auditory system, one can use techniques that alter the linguistic content of speech. Distortions to the speech signal can consequently also affect the linguistic message conveyed (31, 32). These findings show that the envelope and the linguistic content of speech are interdependent (13, 33–35). However, the relative contributions to neural phase-locked responses of the speech envelope on the one hand and the linguistic content of speech, on the other hand, are difficult to disentangle from each other. Several studies have shown the applicability to use amplitude-modulated (AM) stimuli to assess phase-locked responses to envelope modulations (36–39).

Sinusoidally amplitude-modulated (SAM) stimuli are at the basis of the other paradigm to investigate the neural representation of envelope modulations. These stimuli evoke auditory steady-state responses (ASSR) (40) of which the strength reflects the ability of the auditory pathway to phase-lock to the stimulus' modulation frequency (i.e., the response is synchronized to the envelope fluctuations). ASSRs evoked by stimuli with modulations below 20 Hz originate predominately from the auditory cortex, while those evoked with higher frequencies originate from subcortical and brainstem regions (41–44). Studies have indicated that speech perception performance in noise is correlated with 40-Hz ASSRs (45–47) and 80-Hz ASSRs (47–49). In addition, ASSRs elicited by 20-Hz and 4-Hz modulations are associated with phoneme and sentence scores, respectively (48–50). To obtain a sense of the overall capacity of neural modulation processing, ASSRs are measured over a wide range of modulation frequencies. The ASSR amplitude as a function of modulation frequency is the temporal modulation transfer function (TMTF). The TMTF shows a broad peak around 80 and 40 Hz (36–39), and also around 20 Hz (36). Interestingly, the TMTF shows large variations in ASSR evoked by modulation frequencies below 20 Hz and across listeners (36). Therefore, to gain insight into the overall processing capacity of these slow modulations, one would have to measure several ASSRs within this range to evaluate the overall capability to process speech-relevant modulations. However, this approach is time-consuming and could potentially be performed more efficiently using a speech-like stimulus that contains the modulation frequencies of interest.

To overcome the issues that are encountered with speech and SAM stimuli, Gransier and Wouters (51) developed the Temporal Envelope Speech Tracking (TEMPEST) framework. The TEMPEST framework enables the creation of stimuli with parameterized envelopes which can be used to assess the effect of specific characteristics of the speech envelope on neural processing (e.g., envelopes that contain the same modulations as natural speech). In the present study, we investigate whether TEMPEST-based stimuli that consist of syllabic-like and phonemic-like modulations—as present in natural speech—can be used to gain insight into the speech-weighted electrophysiological TMTF of normal-hearing listeners. To this end, we elicited responses with TEMPEST stimuli based on distributions of modulation frequencies close the syllable (~4 Hz) and phoneme (~20 Hz) rates in speech. Furthermore, we also recorded ASSRs, which are normally used to assess the electrophysiological TMTF, with modulation frequencies that covered the same range as those in the TEMPEST stimuli. We compared the overall activity of the TEMPEST neural responses and that of the ASSRs. We expect that the overall TEMPEST neural activity corresponds to the speech-weighted overall activity within the ASSR TMTF and that the TEMPEST framework can be used to efficiently probe the speech-weighted electrophysiological TMTF in normal-hearing listeners. To this end, we used different power- and phase-based electrophysiological metrics that are widely used in the literature. Many studies make use of various electrophysiological metrics (or terminologies) to characterize phase-locked responses to AM stimuli. Some of the studies made use of power-based metrics [e.g., in Gransier et al. (36), Purcell et al. (37), Poulsen et al. (38)] while other studies applied phase-based metrics [e.g., in Luo and Poeppel (17), Howard and Poeppel (18)]. Due to the use of different metrics, comparing results across studies is difficult. Therefore, we included different power- and phase-based metrics and investigated how they relate to each other in order to facilitate these comparisons across studies.

## Materials and methods

### TEMPEST framework

**Figure 1:**
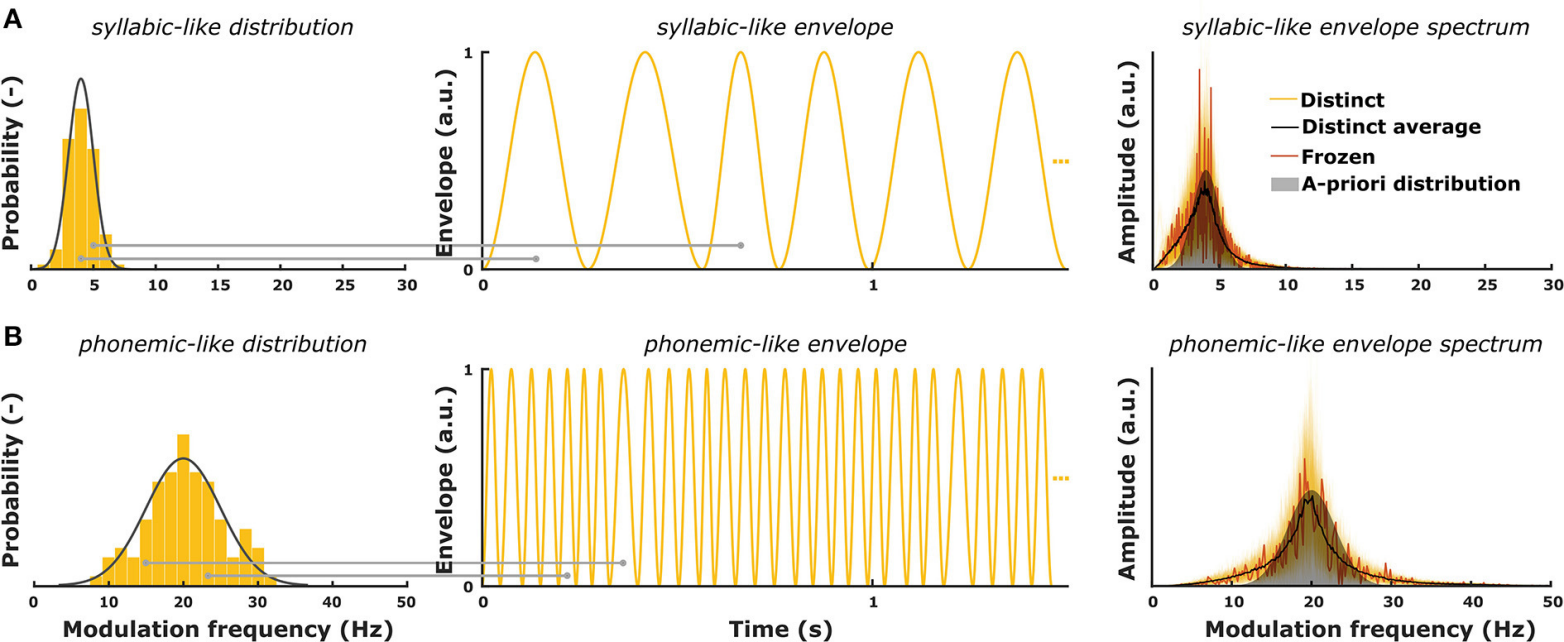
**(A)** A-priori modulation frequency distribution for syllabic-like envelopes (mean = 4 Hz; standard deviation = 1 Hz) and an exemplary envelope. The right panel shows modulation spectra of 200 syllabic-like TEMPEST envelopes (yellow) along with the averaged distinct spectrum (black) and the spectrum of the frozen envelope (red). **(B)** A-priori modulation frequency distribution for phonemic-like envelopes (mean = 20 Hz; standard deviation = 3 Hz) and an exemplary envelope. The right panel shows modulation spectra of 200 phonemic-like TEMPEST envelopes (yellow) along with the averaged distinct spectrum (black) and the spectrum of the frozen envelope (red). Histograms show the sampled modulation frequencies of the exemplary envelopes. Horizontal gray lines depict the sampling of modulation frequency for two envelope windows with corresponding window length (= 1/f_m_).

Gransier and Wouters (51) introduced the TEMPEST framework in which amplitude-modulated stimuli are created based on an a-priori distribution of modulation frequencies that are relevant for speech. The purpose of the TEMPEST framework is to evaluate the overall envelope encoding ability of the auditory system with stimuli containing a range of envelope modulations. TEMPEST stimuli have a quasi-regular envelope which is generated by concatenating windows over time (Figure 1). Each window in the envelope can represent the occurrence of an acoustic unit in natural speech. The duration of each window depends on random sampling from a probability distribution of modulation frequencies. Each randomly sampled modulation frequency (f_m_) is inverted to determine the duration (T_window_ = 1/f_m_) of subsequent windows (Figure 1). Furthermore, each window can have some fixed or variable parameters, such as peak amplitude, onset time, etc. A simple example is the SAM stimulus, which can be created within the TEMPEST framework using sinusoidal windows with a fixed peak amplitude and only one modulation frequency. The next examples are two TEMPEST stimuli used in this study (Figure 1, right). These stimuli have different modulation frequency distributions: one centered around 4 Hz (syllable rate) and one around 20 Hz (phoneme rate) (Figure 1, left). Due to sampling of the distributions, the envelope modulation spectrum will also contain these modulation frequencies with a peak at the center frequency. After its generation, the envelope is used to modulate a carrier signal to finalize the creation of the TEMPEST stimulus.

The main goal of this study is to validate whether the TEMPEST framework can be used to assess the speech-weighted electrophysiological TMTF in normal-hearing listeners. The TEMPEST framework would be a useful tool to investigate the overall neural capability to process envelope modulations which can potentially be related to speech perception performance. To this end, we generated “basic” TEMPEST stimuli using a Gaussian probability function of low modulation frequencies that are apparent in the speech envelope.

### Participants

Ten normal-hearing native-Dutch young adults (ages from 19 to 27 years; 3 males and 7 females) participated in this study. No participants had neurological deficits. All participants had normal hearing (pure tone thresholds ≤ 25 dB HL for all octave frequencies between 250 and 8,000 Hz). This study was approved by the Medical Ethical Committee of the UZ Leuven hospital (study number: B322201524931). All participants gave written informed consent before participation.

### Stimuli

#### SAM stimuli

ASSRs with different modulation frequencies were recorded to obtain individual electrophysiological TMTFs within the modulation frequency ranges of the TEMPEST stimuli. Modulation frequencies of the SAM stimuli were chosen to sample the modulation bands of the TEMPEST stimuli (Figure 1, left). Syllabic-like SAM stimuli with modulation frequencies of 2–6 Hz and phonemic-like SAM stimuli with modulation frequencies of 17–23 Hz were included. All SAM stimuli were created in a custom stimulation software (52). Modulation frequencies were adjusted such that there is an integer number of cycles within one trial of 1.024 s. However, we will further report using rounded modulation frequencies for readability. Modulation depth was set at a maximum of 100% in order to elicit as large ASSRs as possible. The carrier was speech-weighted noise which was generated from the long-term average spectrum of 730 Dutch sentences of the LIST corpus (53). Blocks of 2.56 min were recorded in each measurement session so that 300 trials in total were recorded for each modulation frequency.

#### TEMPEST stimuli

TEMPEST envelopes for this study were generated in Matlab R2016b using Hann windows. Hann windows were used because they have a start- and endpoint at zero to prevent discontinuities in the envelope. The peak amplitude of the windows was always at a maximum of 1 such that the effective modulation depth of the TEMPEST stimuli was 100%. We generated two types of TEMPEST stimuli: syllabic-like and phonemic-like stimuli (Figure 1). Modulation distributions of the TEMPEST stimuli were based on modulation rates that are particularly important for speech, i.e., the natural rates of syllables and phonemes (2, 7). The modulation distribution of syllabic-like TEMPEST envelopes closely matched the low envelope modulation spectrum of speech, which shows a peak around 4 Hz (3, 4). The phonemic-like modulation distribution was based on phoneme length statistics in speech from which the mean duration was found to be around 50 ms (54), which corresponds to a center modulation frequency of 20 Hz. The standard deviations of the distributions were 1 Hz and 3 Hz the envelopes of the syllabic-like and phonemic-like TEMPEST stimuli, respectively (Figure 1).

The duration of the syllabic-like and phonemic-like stimuli were 5.12 s and 25.6 s long in order to reach a similar number of envelope windows and to sufficiently sample the modulation distributions. The envelopes were tested for sufficient statistical similarity to the modulation distribution using the Kolmogorov-Smirnov test with a significance level of α = 0.05. Additionally, we applied criteria to ensure that the envelope modulation sample mean and standard deviation did not deviate too far from those of the a-priori distribution. We used Δμ ≤ 0.05 Hz and Δσ ≤ 0.05 Hz for syllabic-like envelopes, and Δμ ≤ 0.25 Hz and Δσ ≤ 0.1 Hz for phonemic-like envelopes, with Δμ the difference between the means and Δσ the difference between standard deviations of the sample and a-priori distributions. Envelopes that did not meet these criteria were discarded and new ones were generated instead until they met the criteria. This procedure was continued until 200 syllabic-like and phonemic-like TEMPEST envelopes were obtained. Only 20% of the total amount of generated envelopes passed the test and both criteria. Finally, these envelopes were used to modulate segments of speech-weighted noise based on Dutch LIST sentences (53).

In the main experiment, one single syllabic-like and one phonemic-like stimulus were presented repeatedly to the listener. These stimuli are referred to as frozen stimuli since the same temporal pattern was used over again. The goal of the frozen stimuli was to test robust neural phase-locking and evoked power in the modulation distribution frequency range and to compare this neural activity with ASSRs. Additionally, the remaining syllabic-like and phonemic-like stimuli were presented only once to the listener. Since these stimuli were temporally different from each other, they are referred to as distinct stimuli. Distinct stimuli were used as a baseline measurement with respect to the frozen stimuli (17, 55–57). The number of distinct stimuli equaled the number of repeated presentations of the frozen stimulus so that bias by differences in the number of trials is minimized (58). Stimuli were presented in blocks of 5.12 min, in which either frozen stimuli were repeated or distinct stimuli were presented in random order. In total, there were 156 frozen and distinct syllabic-like trials (12 presentations in 13 blocks), and 180 frozen and distinct phonemic-like trials (60 presentations in 3 blocks). Each block was preceded with a short 2.56-s TEMPEST segment generated with the same parameters. The evoked neural activity to this segment contains an onset response that would interfere with the main analysis. Therefore, the EEG recordings corresponding to this segment were immediately discarded.

### Equipment

#### Calibration and presentation setup

Presentation of all stimuli was done using custom-built software interfacing with an RME-Hammerfall DSP Multiface II soundcard and delivered monaurally through an Etymotic ER-3A insert earphone to the right ear. All stimuli were calibrated using a 2-cc coupler of an artificial ear (Brüel & Kjær, type 4,152) and presented at 70 dB sound pressure level (SPL) at a sampling rate of 32 kHz. Two measurement sessions were conducted whereby each session started with a set of ASSR stimuli in a pseudo-random order which was followed by a set of phonemic-like and syllabic-like TEMPEST stimuli in a pseudo-random order as well.

#### EEG recording setup

EEG was recorded using a 64-channel BioSemi ActiveTwo recording system with a sampling rate of 8,192 Hz and a recording bandwidth of 0 to 1,683 Hz. A head cap with 64 Ag/AgCl recording electrodes was placed on the scalp of every participant. The electrode positions were placed across the scalp according to the international standard 10–20 system (59). All recordings were made in a double-walled soundproof booth that is equipped with a Faraday cage to avoid signal interference as much as possible. Participants watched a silent movie by choice while seated in a relaxing chair. They were offered a head pillow and asked to move as little as possible to minimize head movement/muscle artifacts.

### Signal processing and response quantification

#### Preprocessing

**Figure 2:**
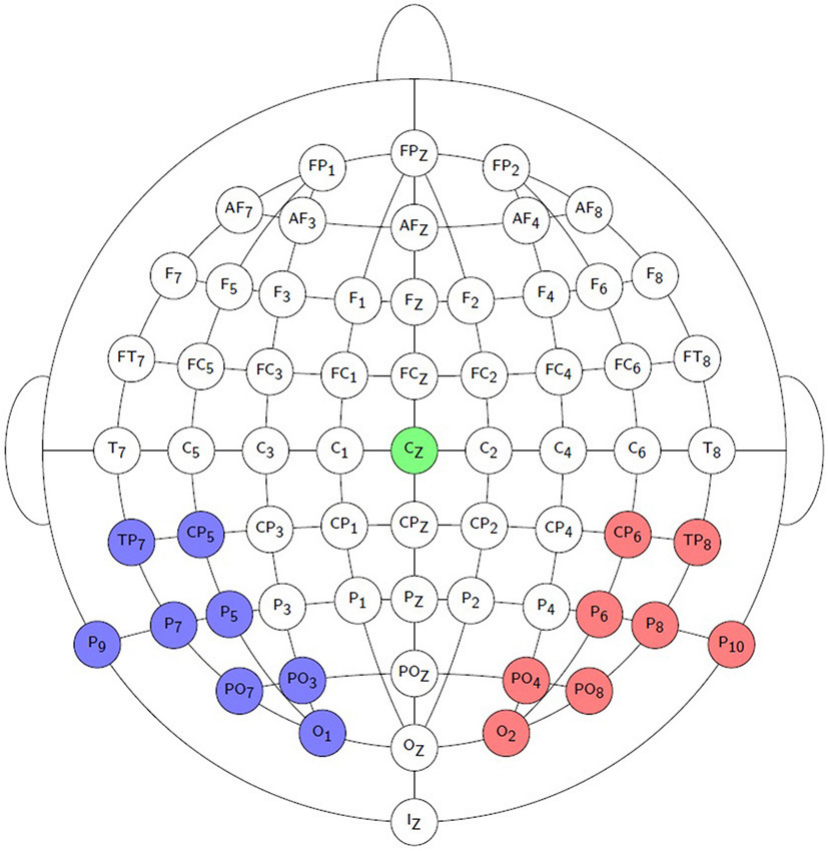
Visualization of the EEG recording electrodes used to form the left channel (blue) and the right channel (red). The reference EEG electrode Cz is indicated by the green color.

Offline signal processing was done in Matlab R2016b. EEG recordings were high-pass filtered using a 1st order Butterworth filter with a cut-off frequency of 0.5 Hz to remove any DC component and slow drifts. Recordings were referenced to electrode Cz by subtracting the recording of Cz from those of the other channels. 5% of the trials were discarded from the analysis based on the highest peak-to-peak amplitudes, as they were assumed to contain muscle and other recording artifacts. Due to measurement errors, not all trials could be obtained from each participant. Only 108 frozen phonemic-like trials could be retained from Participant 7, while 162 phonemic-like trials could be retained in all other cases. The minimum number of retained syllabic-like trials is 115 and the maximum number is 136 across all participants. Time signals of the parieto-occipital recording electrodes were averaged into a left and a right hemispheric channel. Recording electrodes O1, PO3, PO7, P9, P7, P5, CP5, and TP7 formed the left hemispheric channel, while recording electrodes O2, PO4, PO8, P10, P8, P6, CP6, and TP8 formed the right hemispheric channel. See Figure 2 for a visualization of the selected electrodes.

In the case of ASSRs, all 300 trials of each modulation frequency were successfully recorded. Syllabic ASSR (f_mod_ = 2–6 Hz) recordings were grouped into sweeps of 5 trials, while phonemic-like ASSR (f_mod_ = 17–23 Hz) recordings were grouped into sweeps of 1 trial. Syllabic-like and phonemic-like ASSR sweep lengths were thus 5.12 s and 1.024 s, respectively. Consequently, the number of cycles in each sweep is similar for both syllabic-like and phonemic-like ASSRs in order to have similar phase estimation during analysis. The rest of the preprocessing procedure is the same as for the TEMPEST recordings.

#### Neural response analyses

Amplitude and phase for each modulation frequency were extracted from the individual or averaged response trials after transforming into the spectral domain. ASSR sweeps were transformed using the discrete Fourier transform. TEMPEST response trials were transformed into Fourier spectrograms with Hanning windows in which the window length and window overlap were tuned such that phase estimation is similar to that for ASSRs. The window length was equal to the length of the corresponding syllabic-like or phonemic-like ASSR sweep. The window overlap corresponded to three times the reciprocal of the mean modulation frequency in each TEMPEST stimulus such that subsequent windows are, on average, one cycle from each other. Thus, for syllabic-like TEMPEST, spectrograms were computed with 5.12 s window length and 0.25 s window step, whereas for phonemic-like stimuli, a window length of 1.024 s and a window step of 0.05 s were used. Since different spectrogram parameter values were used, the frequency resolution differed between syllabic-like and phonemic-like stimuli. Response bins are 0.195 Hz/bin and 0.977 Hz/bin, respectively. Amplitude and phase were extracted from each time-frequency bin in the spectrogram. These values were used to compute several electrophysiological metrics listed below.

To gain insight into the characteristics and robustness of the recorded neural responses and to compare the TEMPEST responses with ASSRs, four electrophysiological metrics were employed in our analysis. A small selection of metrics have been employed because many different metrics are being used in the literature and this makes comparisons and conclusions across studies more difficult. In order to investigate how different metrics relate to each other and to facilitate comparisons between studies, the metrics used in our analyses represent some of the most widely used ones in the power and phase domain. Two of them are power-based metrics, namely power and signal-to-noise ratio (SNR) of the averaged response. Power is computed after obtaining the amplitude spectrum of the averaged neural response and squaring the amplitude in each frequency bin This metric reflects the overall neural activity evoked by the stimulus (60). The SNR is taken as the power of the averaged neural response divided by power of the neural background noise. Power of the averaged neural response is computed as the mean power across stimulus trials in each frequency bin. Power of the neural background noise is computed as the variance of power across stimulus trials divided by the number of trials in each frequency bin. This estimation of neural background noise is more viable for TEMPEST responses than the estimation from neighboring noise bins which is commonly used in case of ASSRs (40). This is because TEMPEST responses are expected to contain evoked power within a certain frequency band whereas ASSRs only have evoked power in the modulation frequency bin. Additionally, as the neural background noise typically exhibits a 1/f spectrum, noise power at the lower frequency side is higher than at the higher frequency side. Evoked responses to a repeated stimulus expected to be consistent in power and phase across trials, while neural background noise adds a random amplitude and phase to that of the evoked response in each trial. Under this assumption, variance in power across trials divided by the number of trials reflects neural background noise power (61). The two power-based metrics are ubiquitously used in the neuroscience field to indicate the strength and quality of the measured averaged response. The other two metrics are solely based on the phase of the individual response trials: inter-trial phase coherence (ITPC) and pairwise phase consistency (PPC). The first metric, ITPC, indicates consistency of phase-locking to a stimulus based on the magnitude of the average of unit vectors rotated by extracted phases θ_n_ across N trials (17, 62).


(1) $$\begin{array}{l} ITPC\;=\;{\left(\overset{N}{\underset{n=1}{\sum }}\frac{\cos\theta_{n}}{N}\right)}^{2}+{\left(\overset{N}{\underset{n=1}{\sum }}\frac{\sin\theta_{n}}{N}\right)}^{2} \end{array}$$

The ITPC is commonly used to investigate robustness of phase-locking with different stimulus parameters (17, 18, 55, 57, 62–64). However, despite its considerable presence in the literature, the ITPC is biased by the number of trials with fewer trials resulting in a larger positive bias in the outcome. This bias could hamper comparison between conditions and/or studies with different amounts of trials (58, 61, 65). In contrast to ITPC, the PPC is an unbiased estimate of phase-locking because it is based on the averaged dot product of all possible phase pairs θ_n_ and θ_m_ across *N* trials (66).


(2) $$PPC\;=\;\frac{2}{N(N-1)}\sum_{n=1}^{N-1}\sum_{m=n+1}^{N}cos(\theta_{n}-\theta_{m})$$

When phase consistency is high, then distances between phase pairs will become smaller and thus dot products will be larger. The advantage of the PPC is that it allows for comparison between studies and conditions even with different trial numbers. Both ITPC and PPC take up values between 0 and 1, with 0 indicating no phase-locking at all and 1 indicating perfect phase-locking across trials. Note that ITPC and PPC for TEMPEST responses are computed for each time and frequency bin. In order to obtain electrophysiological patterns as a function of modulation frequency in each participant, results of each metric were averaged in the time domain.

Responses were tested for significance against the neural background noise using the Hotelling T^2^ test (52, 67). ASSRs were tested only at their modulation frequency bin while TEMPEST neural activity was tested in each modulation frequency bin of the spectral domain. To evaluate similarity between ASSR patterns and between TEMPEST patterns measured with different electrophysiological metrics and whether different metrics would reveal different characteristics of the neural patterns, the patterns were subjected to correlation analyses. Only significant response bins were included in the analyses. Additionally, because ITPC and PPC are bounded between 0 and 1, their values were first transformed using the Fischer z-transformation. Pearson's correlation coefficients and corresponding *p*-values were then reported. The significance level was α = 0.05 at all times and *post-hoc* Bonferroni correction was used to control for false discovery rate since multiple correlations were being tested simultaneously.

#### Comparison between TEMPEST and ASSR TMTF patterns

**Figure 3:**
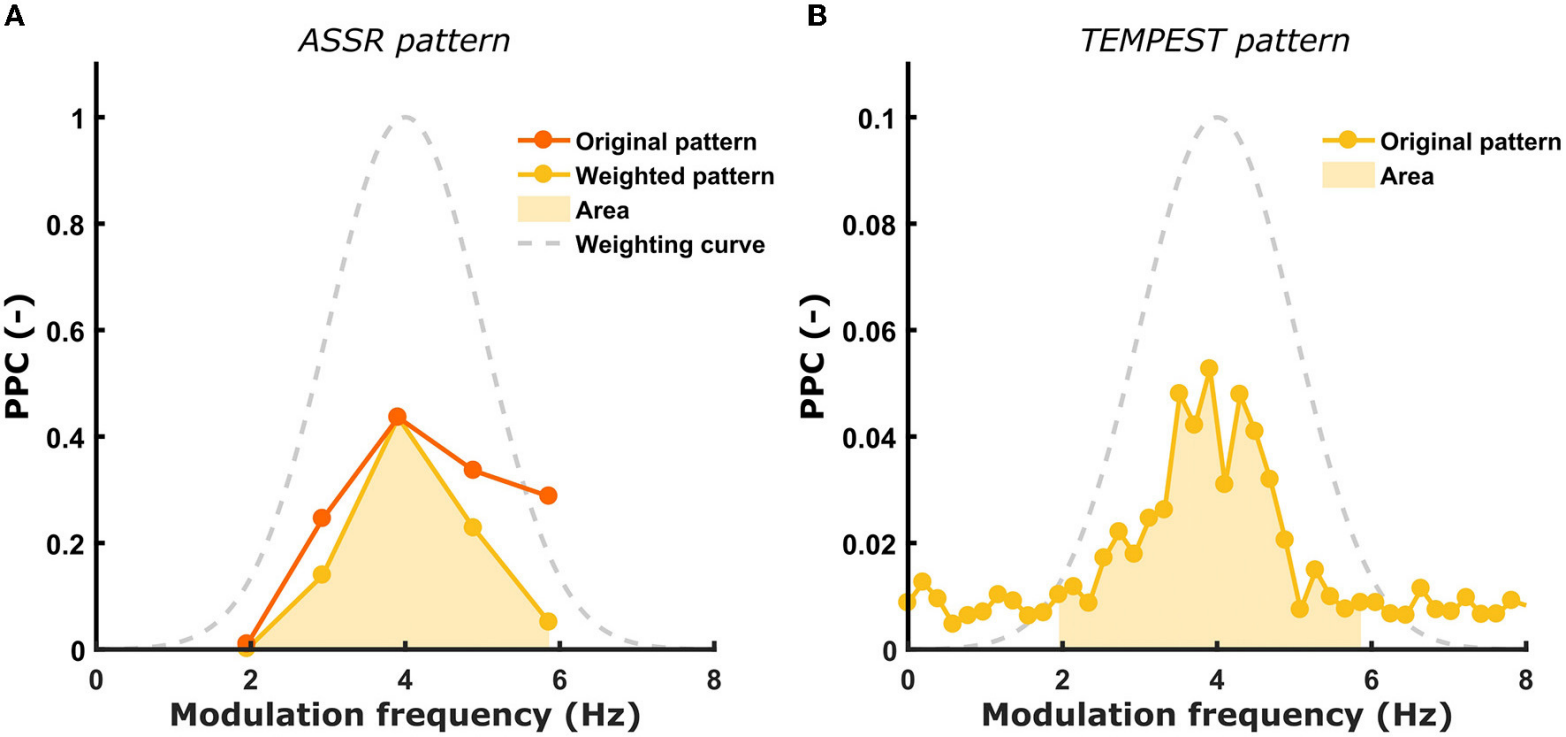
Illustration of ASSR pattern weighting and computation of the area under the curve. **(A)** the original ASSR pattern is weighted by the Gaussian modulation distribution of the corresponding TEMPEST stimuli. The computed area under the weighted ASSR pattern is depicted as the shaded area. **(B)** The computed area under the TEMPEST pattern is depicted as the shaded area. Syllabic-like pattern data came from the left hemispheric channel in Participant 3.

The main goal of this study is to investigate whether the neural activity evoked by TEMPEST stimuli is comparable to the overall ASSR activity (i.e., the TMTF) in the same frequency band. Usually, the TMTF is obtained by setting out ASSR amplitude as a function of modulation frequency (36–39). However, in this study not only ASSR patterns of power, but also of SNR, ITPC and PPC were used. When the ASSR TMTF shows a prominent peak, we hypothesize that the TEMPEST neural activity would also show a relatively large peak and vice versa for each metric. The presence of prominent peaks translates into a larger area under the TMTF pattern. To compare the TEMPEST patterns with those of ASSRs, areas under patterns of the same metric were computed and correlated with each other across all participants in the left and right hemispheres. Before computing the area of ASSR patterns, patterns were first weighted according to the corresponding TEMPEST modulation distribution in order to account for the relative contribution of each modulation frequency to the TEMPEST neural activity. Each modulation frequency of the SAM stimuli contribute equally to the ASSR patterns. However, these contributions are not equal anymore in case of TEMPEST due to the a-priori modulation frequency distribution used to generate the stimuli. To achieve this weighting of the ASSR pattern, it is multiplied with the Gaussian curve of the corresponding syllabic-like or phonemic-like TEMPEST modulation distribution. By doing this, the TEMPEST and ASSR neural evoked activity can be directly compared to each other after accounting for the modulation distribution shape. Areas under the patterns were computed between 2 and 6 Hz for syllabic-like responses, and between 17 and 23 Hz for phonemic-like responses (Figure 3).

The area was computed by summing up the values in each frequency bin within the restricted band. Finally, to test the relative correspondence between the ASSR and TEMPEST patterns, Pearson's correlations between the TEMPEST and ASSR areas across participants were computed. Only areas of the same metric from ASSR and TEMPEST analyses were correlated (e.g., the area of ASSR SNR was correlated with the area of TEMPEST SNR). Partial Pearson's correlations were computed between TEMPEST and ASSR power area in order to control for any potential effects of induced power area. Induced power is the power that appears in the EEG in any frequency band while listening to a stimulus. In order to investigate whether neural phase-locking to the TEMPEST and SAM stimuli correspond to each other, the correlation with induced power must be controlled for. The induced power area in the syllabic-like frequency range was computed from the averaged power spectrum of the distinct phonemic-like TEMPEST stimuli, whereas the induced power area in the phonemic-like frequency range was computed from the distinct syllabic-like TEMPEST stimuli. The significance level for the correlations was α = 0.05 and *p*-values were corrected with the Bonferroni procedure.

## Results

### Evaluation of electrophysiological metrics

#### ASSR

**Figure 4:**
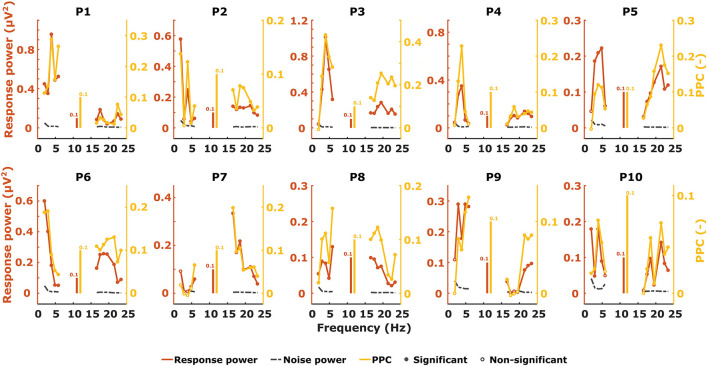
Unweighted ASSR power (red), noise (black), and PPC (yellow) patterns from the left hemispheric channel as a function of modulation frequency.

We measured ASSRs with 2–6 Hz (syllabic-like) and 17–23 Hz (phonemic-like) modulation frequencies and obtained the response pattern across modulation frequency for each participant and electrophysiological metric, which is very similar to how TMTFs are obtained elsewhere. Almost all ASSRs were found to be statistically significantly different from noise using the Hotelling T^2^ test. Figure 4 shows the individual ASSR patterns measured with response power and PPC for syllabic ASSRs in the left hemispheric channel. In this case, the patterns of these two metrics are relatively similar to each other within each participant. The different shapes of the patterns demonstrate the large variability in ASSRs across modulation frequency and participants.

**Figure 5:**
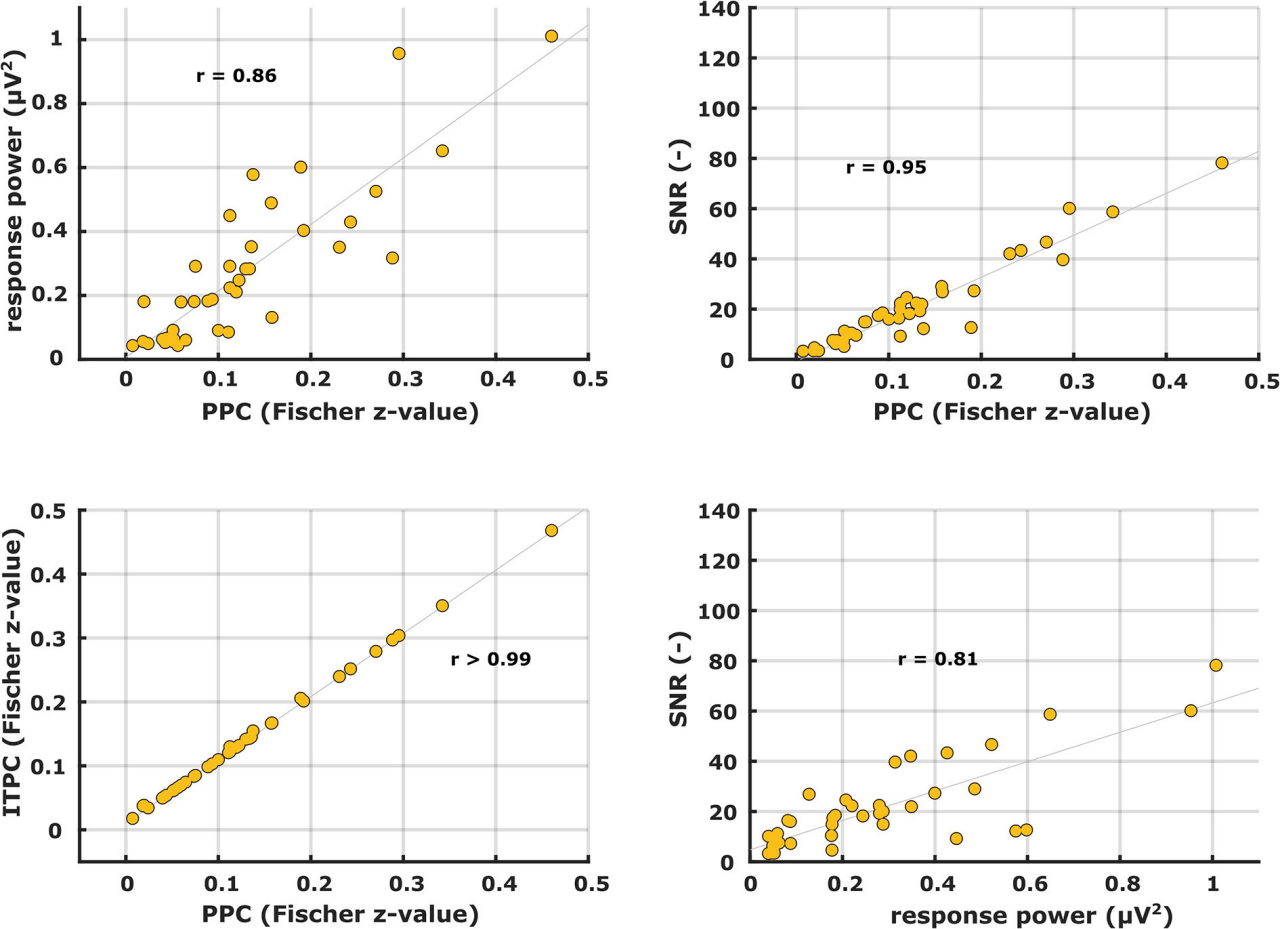
Scatter plots between different ASSR electrophysiological metrics for syllabic-like stimuli in the left hemispheric channel.

Patterns of the other electrophysiological metrics are not shown but their similarity in shape to each other was evaluated with correlation analyses. Examples of correlation scatterplots for the syllabic-like responses in the left hemispheric channel are shown in Figure 5. Table 1 summarizes all Pearson's correlation coefficients between the different electrophysiological metrics. Since the response power and PPC patterns were relatively similar, they were highly correlated with each other [*r* (38) = 0.85, *p* < 0.0001]. The ITPC and PPC showed an almost perfect linear correlation (Figure 5, bottom left) based on the fact that the PPC is an unbiased estimate of phase-locking compared to the biased ITPC due to the number of trials. Exchanging the ITPC for PPC would not virtually change the interpretation of the results. The next highest correlations were found between SNR and PPC, which are very high [from *r* (38) = 0.92 to *r* (64) = 0.99, *p* < 0.0001]. Comparing the ASSR power with SNR and PPC resulted in moderate to high correlation coefficients. Each correlation coefficient was found to be highly significant (Table 1).

**Table 1:** Pearson's correlation coefficients between different electrophysiological metrics of syllabic-like and phonemic-like ASSRs in the left and right hemispheric channels.

	**Syllabic-like**		**Phonemic-like**	
	**Left**	**Right**	**Left**	**Right**
	***(df =*** **37)**	***(df =*** **40)**	***(df =*** **64)**	***(df =*** **63)**
Power vs. PPC	0.86^*^ (<0.0001)	0.74^*^ (<0.0001)	0.69^*^ (<0.0001)	0.62^*^ (<0.0001)
SNR vs. PPC	0.95^*^ (<0.0001)	0.92^*^ (<0.0001)	0.98^*^ (<0.0001)	0.99^*^ (<0.0001)
ITPC vs. PPC	> 0.99^*^ (<0.0001)	> 0.99^*^ (<0.0001)	> 0.99^*^ (<0.0001)	> 0.99^*^ (<0.0001)
SNR vs. power	0.81^*^ (<0.0001)	0.63^*^ (<0.0001)	0.68^*^ (<0.0001)	0.63^*^ (<0.0001)

*Corresponding p-values are reported between parentheses*.

* *Significant after post-hoc Bonferroni correction*.

#### TEMPEST

**Figure 6:**
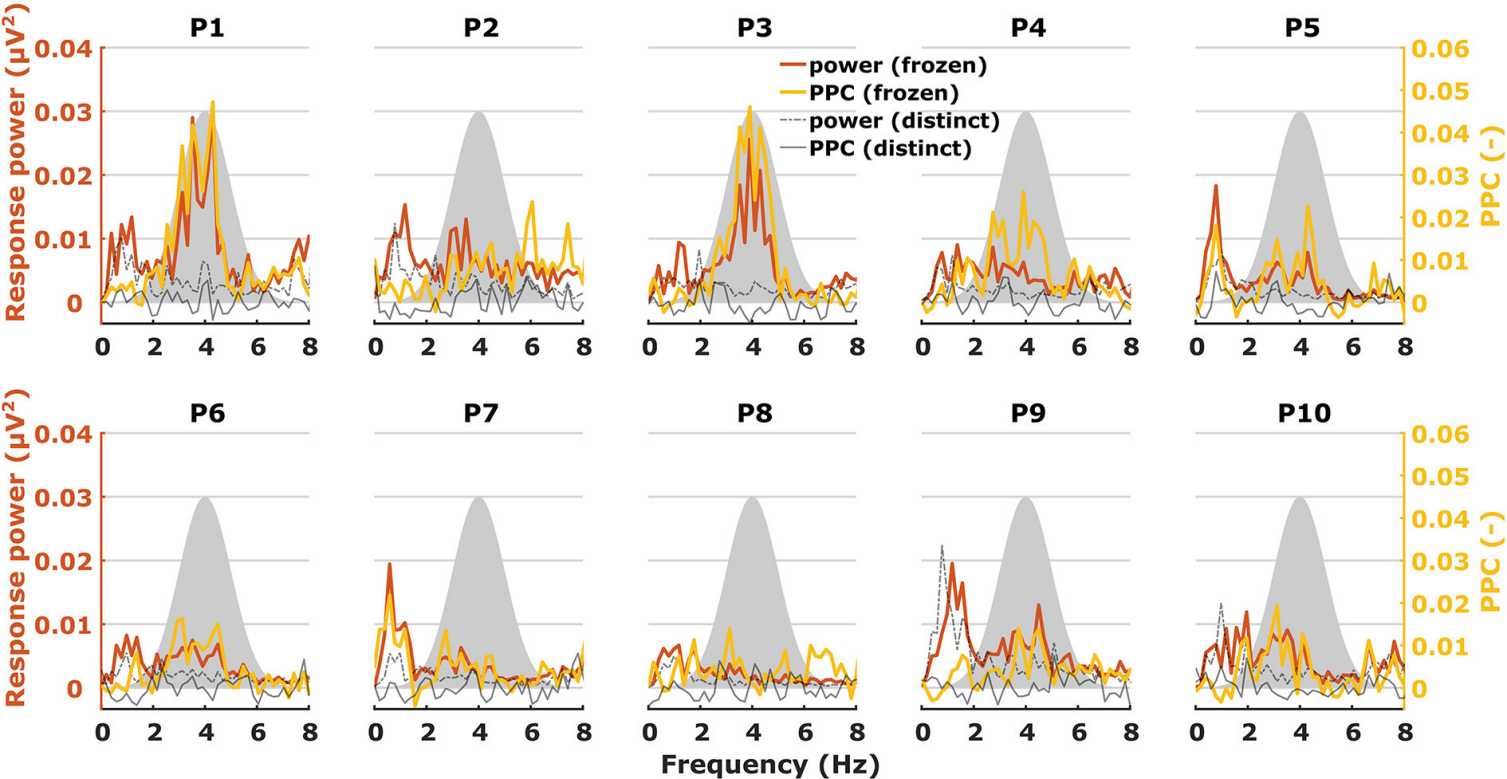
Two different patterns, one of power (red) and one of PPC (yellow), plotted over each other across modulation frequency. The patterns are from TEMPEST syllabic-like responses in the left hemispheric channel for each participant. Black patterns describe the baseline from distinct responses. The shaded Gaussian curve represents the modulation frequency distribution of the stimuli (scaled arbitrarily).

**Figure 7:**
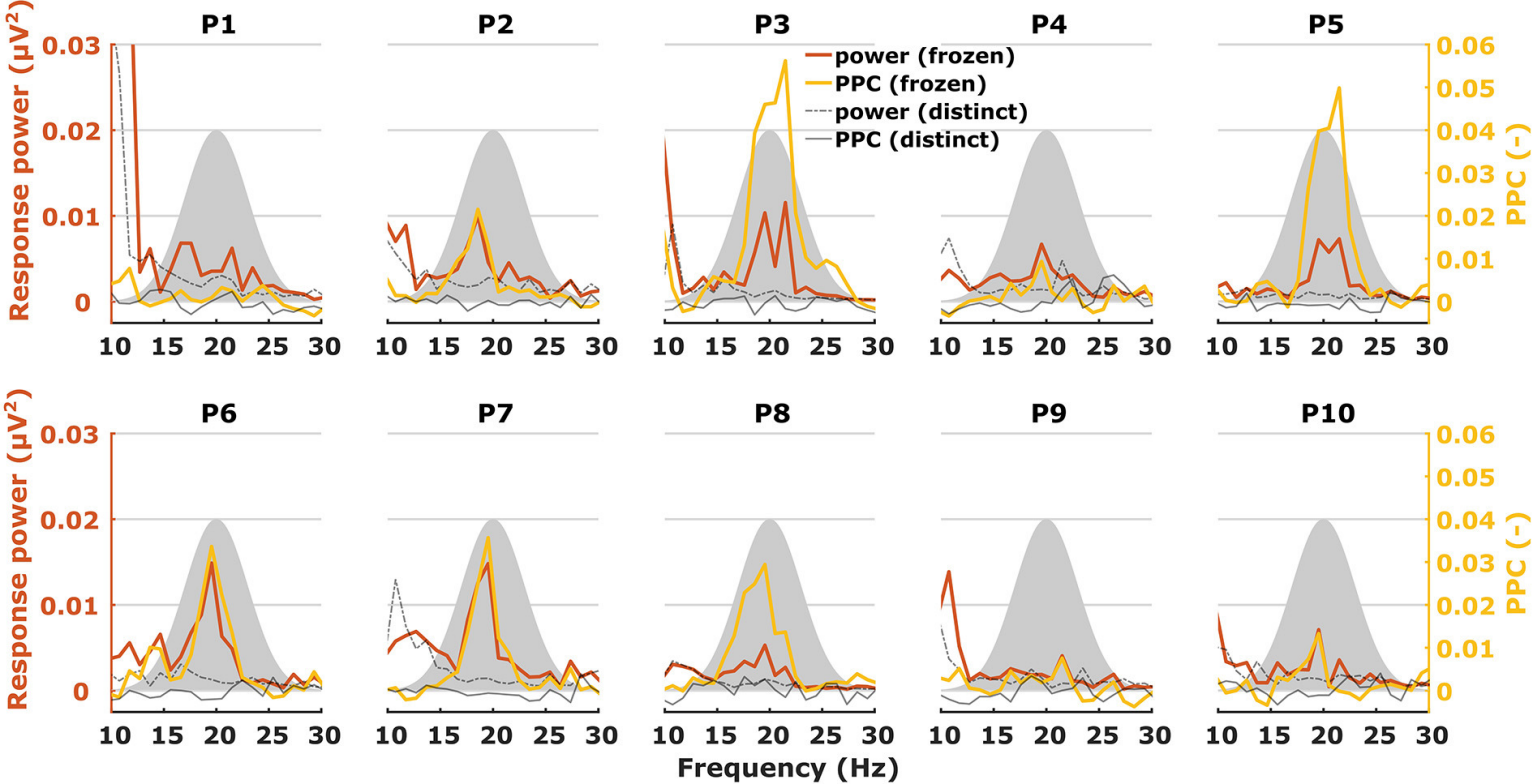
Two different patterns, one of power (red) and one of PPC (yellow), plotted over each other across modulation frequency. The patterns are from TEMPEST phonemic-like responses in the left hemispheric channel for each participant. Black patterns describe the baseline from distinct responses. The shaded Gaussian curve represents the modulation frequency distribution of the stimuli (scaled arbitrarily).

When characterizing neural responses to TEMPEST stimuli for each modulation frequency, all electrophysiological metrics showed variation across participants. Figures 6, 7 show only response power and PPC patterns layered over each other for syllabic-like and phonemic-like neural responses, respectively. Patterns of the other metrics are not shown, but pattern correlations between all four metrics are presented in Table 1. In some participants, distinctive peaks around the mean modulation frequency of the envelope were found in their patterns. For example, participants 1, 3, 5, and 9 showed increased activity around 4 Hz with syllabic-like stimuli. Interestingly, unlike these participants, participant 2 did not show peak activity around 4 Hz but a broader one around 7–8 Hz with syllabic-like stimuli, which corresponds to the range of second harmonic frequencies. With phonemic-like stimuli, participants 3, 5, 6, 7, and 8 showed highly prominent peaks around 20 Hz. As expected, responses to distinct stimuli did not show the increased averaged neural activity as with frozen stimuli.

**Figure 8:**
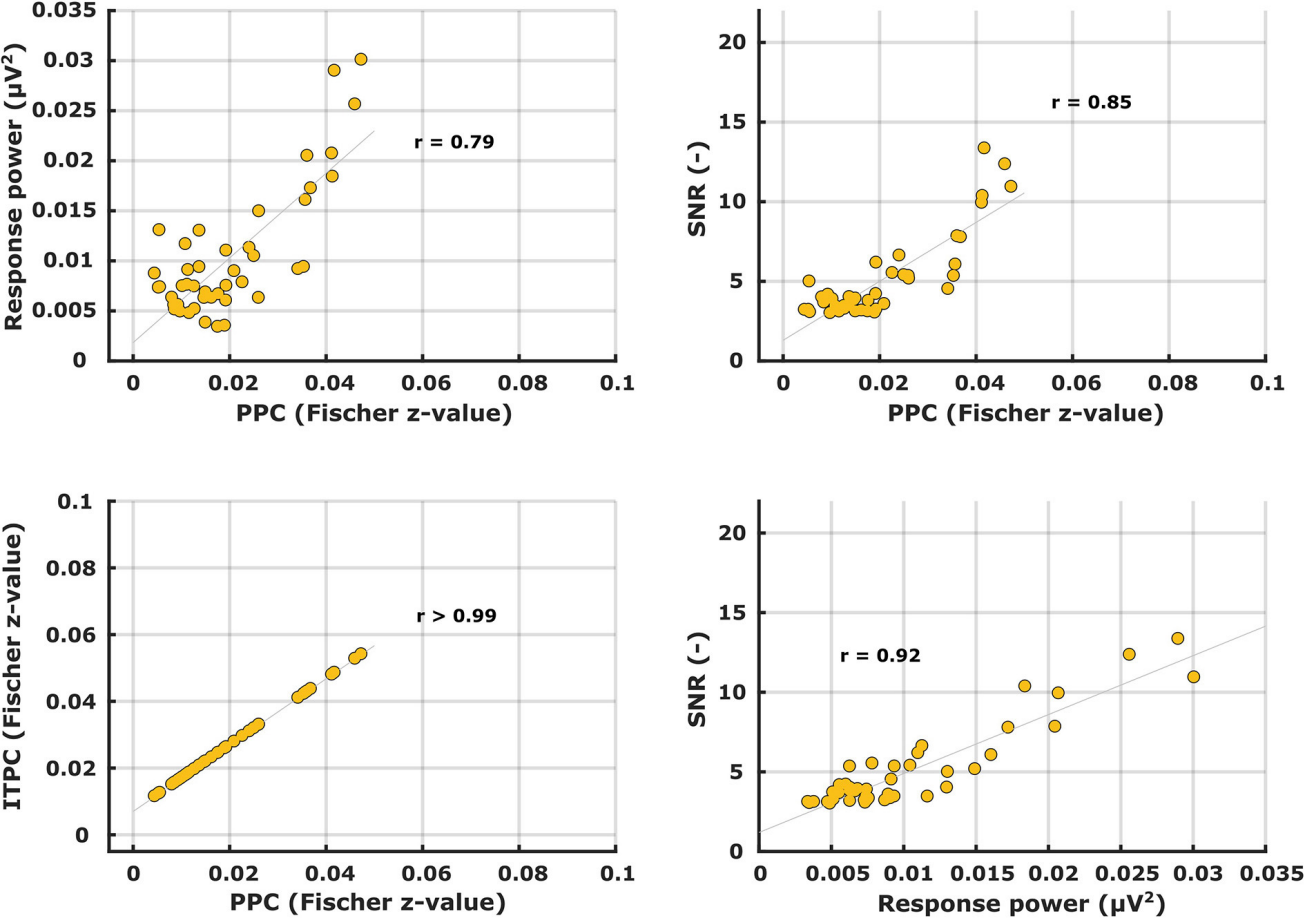
Scatter plots between different electrophysiological metrics for syllabic-like TEMPEST neural responses in the left hemispheric channel.

Patterns of the other metrics are not shown but – like with the ASSRs – their similarity to the PPC patterns was evaluated with correlation analyses. Correlations between the different electrophysiological metric patterns are shown in Table 2. Again, unsurprisingly, the ITPC and PPC showed an almost perfect linear correlation (*r* > 0.99) (Figure 8, bottom left). Exchanging the ITPC for PPC would not virtually change the interpretation of the results as well in this case. Other metric comparisons resulted in moderate to high correlations except for power vs. PPC in the left hemisphere for phonemic-like stimuli. Correlations with PPC for power and SNR were not as strong as those for ASSRs. Each correlation coefficient was found to be highly significant, except for power vs. PPC in the left hemisphere for phonemic-like stimuli (Table 2).

**Table 2:** Pearson's correlation coefficients between different electrophysiological metrics of syllabic-like and phonemic-like TEMPEST neural responses in the left and right hemispheric channels.

	**Syllabic-like**		**Phonemic-like**	
	**Left**	**Right**	**Left**	**Right**
	***(df =*** **42)**	***(df =*** **39)**	***(df =*** **22)**	***(df =*** **27)**
Power vs. PPC	0.79^*^ (<0.0001)	0.49^*^ (0.0012)	0.31 (0.14)	0.44 (0.017)
SNR vs. PPC	0.85^*^ (<0.0001)	0.70^*^ (<0.0001)	0.86^*^ (<0.0001)	0.91^*^ (<0.0001)
ITPC vs. PPC	>0.99^*^ (<0.0001)	>0.99^*^ (<0.0001)	>0.99^*^ (<0.0001)	>0.99^*^ (<0.0001)
SNR vs. power	0.92^*^ (<0.0001)	0.70^*^ (<0.0001)	0.49 (0.015)	0.54^*^ (0.0025)

*Corresponding p-values are reported between parentheses*.

* *Significant after post-hoc Bonferroni correction*.

### Comparison of TEMPEST and ASSR neural activity

**Figure 9:**
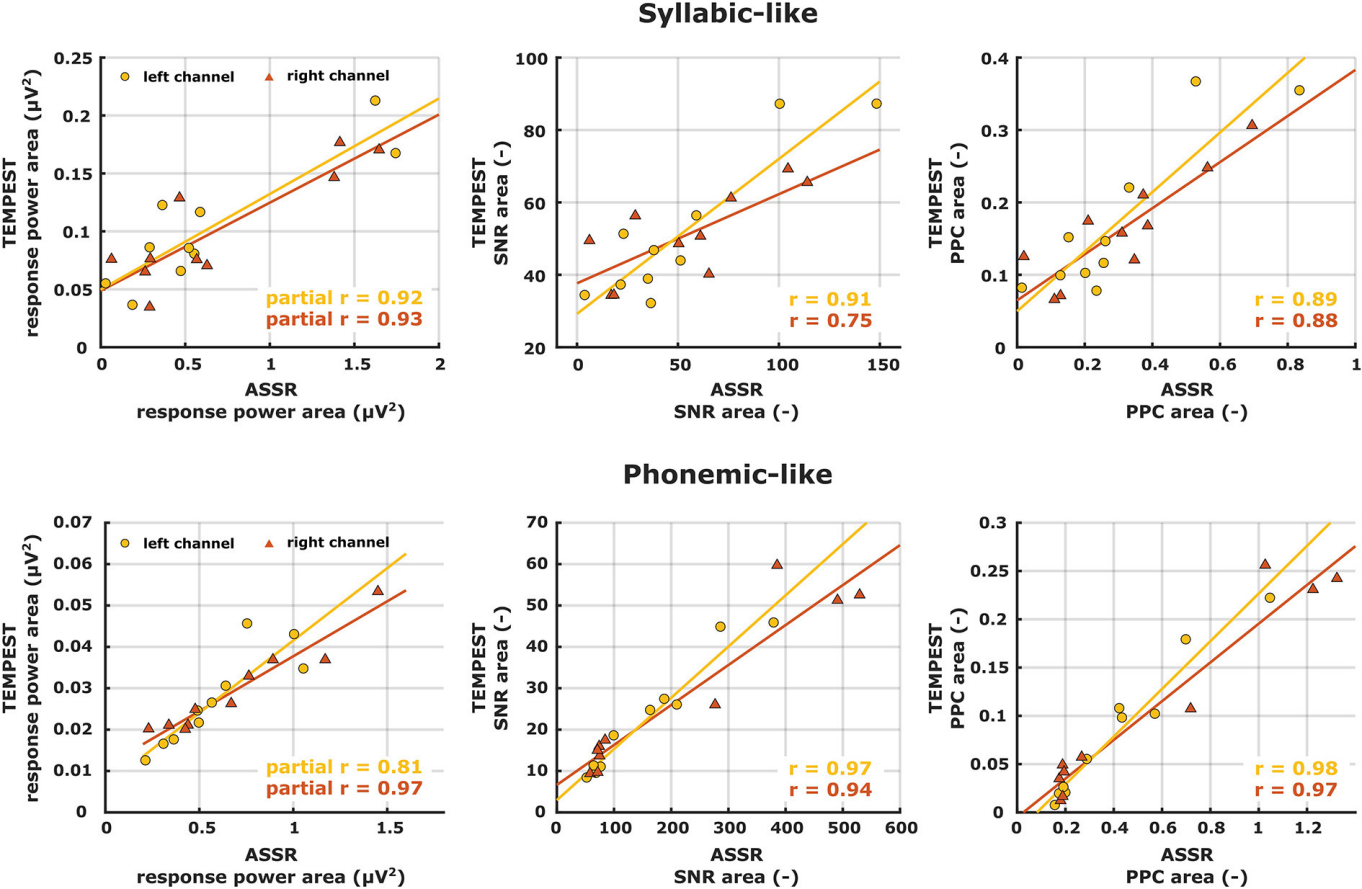
Correlation scatterplots between ASSR and TEMPEST area under patterns of the same electrophysiological metric for syllabic-like (first row) and phonemic-like (second row) neural responses. Pearson's partial correlation coefficients are shown only for the power metric whereas Pearson's correlation coefficients are shown for the other metrics in the right bottom corner.

If an individual ASSR TMTF does not show a prominent peak, then we expect that the TEMPEST neural pattern would not show a peak as well and vice versa. To assess whether the overall activity of ASSR TMTFs corresponds to the activity of TEMPEST responses within participants, we computed the area under the patterns and performed correlation analyses. Only areas under the ASSR and the TEMPEST pattern of the same electrophysiological metric were used because comparing areas with different metrics would not be insightful (e.g., area under ASSR PPC pattern vs. area under TEMPEST SNR pattern). The computed areas were directly correlated across all ten participants for SNR and PPC of syllabic-like and phonemic-like responses in the left and right hemispheres separately (Figure 9, middle and right columns). Based on the almost perfect correlation between ITPC and PPC (Tables 1, 2), ITPC was left out because it would produce the same results as the PPC. All correlation coefficients were found to be strong [*r* (8) = 0.75–0.98] and highly significant after *post-hoc* Bonferroni correction (*p* ≤ 0.001), except for the correlation coefficient between ASSR and TEMPEST SNR area for syllabic-like responses in the right hemispheric channel which was not significant anymore after *post-hoc* correction (*p* = 0.013). For the power metric, partial Pearson's correlations between TEMPEST power area and ASSR power area were computed in order to control for any potential effects of induced power area. Partial correlation coefficients were found to be strong [*r* (7) = 0.81–0.97] and highly significant (*p* ≤ 0.001). These high correlations indicate that the overall activity of the TEMPEST responses corresponds to the speech-weighted overall activity of the ASSR TMTF.

## Discussion

The TEMPEST framework was introduced by Gransier and Wouters (51) to provide an efficient method to investigate the neural representation of the stimulus' envelope with speech-like modulation frequencies. In this study, we aimed to demonstrate a proof-of-concept of the TEMPEST framework to efficiently assess the overall capability of temporal envelope encoding in the auditory pathway. To this end, we investigated whether the neural activity evoked by TEMPEST stimuli corresponds to the speech-weighted electrophysiological TMTF, which is classically measured with ASSRs. We used four different electrophysiological metrics to characterize the neural responses. Two metrics were purely based on power (evoked power and SNR) and two other metrics were purely based on phase (ITPC and PPC) of the individual trials or the averaged trial of the neural response. These metrics were computed for each modulation frequency to obtain neural activity patterns as a function of modulation frequency. This approach is similar to how TMTFs were obtained in other studies using ASSR amplitude (36–39). Comparing the overall neural activity pattern obtained with TEMPEST to the speech-weighted TMTF obtained with ASSRs allowed us to investigate whether they correspond to each other across listeners.

First, we compared neural activity patterns of different metrics with each other for the ASSRs and the TEMPEST responses separately. This is to investigate whether different metrics would reveal different characteristics of the evoked neural activity. A notable case is the almost perfect linear correlation between the ITPC and PPC (Figures 5, 8, bottom left panel) because these two metrics are similar to each other except for a bias due to the number of trials in the ITPC (66). Small deviations occurred because the number of trials was slightly different which resulted in a slightly different bias in ITPC. Furthermore, the bias was relatively small because considerable numbers of trials were used to compute the ITPC (58, 65). Consequently, ITPC results can be exchanged by PPC results without loss of interpretation. As indicated by the high and significant correlation coefficients (Table 1), all individual ASSR TMTF patterns had the same characteristics regardless of the metric used. In the case of TEMPEST activity, syllabic-like patterns of all different metrics significantly correlated with each other. However, phonemic-like patterns of power and PPC did not correlate significantly with each other in both hemispheric channels, and neither did the SNR and power patterns in the left channel (Table 2). Interestingly, the power-based SNR was highly correlated with the phase-based PPC for both ASSRs and TEMPEST responses. While SNR and PPC are based on two independent aspects of the response, i.e., power and phase, the high correlation might be explained by better representation of the phase pattern of the recorded responses due to higher SNR (55, 57). The power metric leads to mostly moderate correlations for both ASSR and TEMPEST stimuli. However, power by itself doesn't tell much about the presence of a response compared to the presence of background noise, which the SNR and PPC can do to a certain extent. This interaction might explain the smaller correlations between power and the other two metrics. Nevertheless, the high correlations also indicate a high similarity of the intersubject variability in envelope modulation processing across modulation frequency across metrics. For example, if a participant showed a large peak of SNR at a certain modulation frequency, then a large peak of PPC is also expected to appear at the same modulation frequency (Figures 5, 8). Therefore, patterns obtained with different electrophysiological metrics, are comparable.

Patterns obtained with TEMPEST stimuli differed across participants which is consistent with the notion that neural phase-locked activity varies considerably across individuals (36). Participants 1, 3, 5, and 9 had relatively large neural activity peaks around 4 Hz when listening to syllabic-like stimuli, while others showed less prominent or no peaks at all. Participants who had prominent syllabic-like neural activity do not necessarily have prominent phonemic-like neural activity as well (e.g., participant 1 in Figures 6, 7), demonstrating the variability across modulation frequency as well (36). Interestingly, participant 2 had no prominent neural activity around 4 Hz when listening to syllabic-like stimuli, but it was instead shifted up to around 8 Hz. One likely explanation is that the higher harmonics of the envelope modulations were preferentially encoded and/or processed in the auditory system in this participant, which is more likely for such slow modulation frequencies (68, 69).

Some studies used non-speech stimuli with different irregular envelope characteristics and investigated their evoked response using phase coherence metrics (55, 57). Both studies of Teng and colleagues used stimuli with dynamic acoustic changes that occur at timescales similar to our stimuli. They used several different stimuli with dynamics at different timescales, some of which coincided with those of syllables and phonemes in speech. Two of those stimuli were the theta- and gamma-sounds. The theta-sound contained changes at a mean timescale of 190 ms (~5 Hz modulation rate) which approximately corresponds to the syllable mean modulation frequency of our syllabic-like TEMPEST stimuli. Similarly, the gamma-sound was temporally related to the phoneme rate with a mean timescale of 27 ms (~37 Hz modulation rate). The authors computed the ITPC of the brain's response for each modulation frequency [note that they used the formula from Lachaux et al. (70), not formula (1) in this study]. Responses evoked by theta sounds showed significantly increased ITPC around 4 Hz and those evoked by gamma sounds around 37 Hz. The peaks that we found in the neural patterns within the modulation frequency range of the TEMPEST stimuli are reminiscent of this finding. Teng and Poeppel (57) also included beta-sounds with mean timescales of 62 and 41 ms (modulation rates of ~16 and ~24 Hz, respectively), thus these stimuli are temporally more closely related to our phonemic-like stimuli. However, they reported a considerable decrease in ITPC with beta sounds compared to theta and gamma sounds. In contrast, we did not find a decrease in ITPC and PPC with phonemic-like TEMPEST stimuli compared to syllabic-like TEMPEST stimuli, and similar conclusions can also be made in the case of response power (Figures 6, 7). Another study by Teng and colleagues used complex stimuli with irregular 1/f modulation spectra (56). They investigated robustness of neural phase-locking by comparing ITPC results with frozen and distinct stimuli (*n* = 25). To this end, the ITPC of the distinct stimuli was subtracted from the frozen ITPC. In a way, this is subtracting the bias from the frozen ITPC and this would be comparable to the PPC. The ITPC difference that they found was at approximately 0.06 in the delta and theta band, which is in line with our syllabic-like results (Figure 6).

Luo and colleagues have also looked at the difference in ITPC between responses evoked by the same (frozen) spoken sentence and responses evoked by different (distinct) sentences (17, 62). ITPC differences of responses to spoken sentences in the delta-theta band are comparable to our syllabic-like PPC results. Another study used mutual information to investigate how much the response phase in the theta band encodes information about the sentence stimulus (71). Peaks of mutual information in the theta band varied across participants, which is in line with the variability in ASSR TMTF for low frequencies (36) and with our results that show variable peaks of activity using syllabic-like stimuli. Additionally, small peaks of mutual information were present in the 22–27 Hz range in some participants and were slightly visible in the grand-average pattern. This frequency range is close to the modulation frequency range of our phonemic-like stimuli. Furthermore, the difference in order of magnitude in mutual information between the theta band and the 22–27 Hz range is similar to the difference that our results exhibit between the syllabic-like and phonemic-like responses. This similarity should be treated with caution because our metrics are not related to mutual information. One thing to keep in mind is that sentence stimuli contain a much wider range of modulation frequencies than our syllabic-like and phonemic-like TEMPEST stimuli.

The main goal of the study was to evaluate whether the global neural activity evoked by TEMPEST stimuli was qualitatively comparable to the speech-weighted overall activity in the electrophysiological TMTF measured with ASSRs. To this end, we computed the area under the patterns of power, SNR, and PPC as a function of modulation frequency of TEMPEST responses and area under the ASSR TMTFs by summing up the values at significant response frequency bins. Before the computation of the area, TMTFs were first weighted with the Gaussian curve of the modulation frequency distribution from the corresponding TEMPEST stimuli. We then computed same-metric correlation coefficients between these areas across participants. All correlations between ASSR and TEMPEST were found to be strong and significant except for the SNR in the right hemispheric channel (Figure 9). These significantly high correlations indicate that the neural activity evoked by TEMPEST stimuli is comparable to those of the speech-weighted TMTF measured with the classical ASSR paradigm. Furthermore, they also show that the variability in the global neural patterns across listeners as measured with TEMPEST stimuli is similar to that found with ASSR TMTFs, which is consistent with the findings by (36). Consequently, evoked TEMPEST responses characterized by any of the three metrics (power, SNR, or PPC) can be used as an indicator of individual neural temporal processing capability within the modulation frequency band of interest.

Although our approach of computing the area under the patterns of TEMPEST neural activity and the TMTF does not consider the exact pattern shapes, we found that the overall activity evoked by TEMPEST stimuli strongly corresponds to the overall activity found in the electrophysiological TMTF. This result is a clear indication that the TEMPEST framework has the potential to evaluate temporal envelope processing in the auditory pathway. Furthermore, since TEMPEST stimuli contain a range of envelope modulations as determined by an a-priori modulation frequency distribution, individual distribution-weighted electrophysiological TMTFs can be efficiently determined, which would otherwise be measured by multiple SAM stimuli, as is clear from Figures 6, 7. Further research on variations of TEMPEST stimuli and improvement of the neurophysiological analyses can potentially push the TEMPEST framework to more clinical usability. Moreover, the TEMPEST framework provides many possibilities to generate TEMPEST stimuli that are parameterized, for example, by a modulation frequency distribution, a modulation depth distribution, window shape with optionally varying parameters, etc. Furthermore, the framework also allows for more complex stimuli such as nesting of two or more TEMPEST envelopes (51), which combines multiple TEMPEST stimuli with different modulation frequency distributions into one stimulus. This approach would be comparable to combining multiple SAM stimuli at different carrier frequencies and is commonly used to electrophysiologically determine frequency-specific hearing thresholds in infants (72).

## Conclusion

The TEMPEST framework (51) provides stimuli that evoke neural phase-locked activity with the same characteristics as the electrophysiological TMTF classically measured with ASSRs after weighting by the TEMPEST distribution. Since TEMPEST stimuli contain a range of envelope modulation frequencies in contrast to single-frequency SAM stimuli, they can be used to efficiently probe temporal envelope processing in the auditory pathway. Any of the four electrophysiological metrics (evoked power, SNR, ITPC, or PPC) can be used to evaluate the degree of neural tracking to amplitude-modulated stimuli. Moreover, TEMPEST stimuli that contain speech-like modulations (such as the syllable and the phoneme rate in speech) have the potential to provide a better understanding of the role of neural envelope processing in speech perception. Not only that, but they could also potentially capture differences in temporal envelope processing in different listener groups with different types of auditory processing deficits. Future work would further investigate the potential of the TEMPEST framework using more complex stimuli by varying several other envelope parameters or combining different stimuli into one stimulus with multiple bands of modulation frequencies, and explore different analysis techniques to exploit its full potential in the neuroscientific and audiological fields.

## Data availability statement

The data generated and/or analyzed during the current study are not publicly available for legal/ethical reasons, they can be obtained on reasonable request to the corresponding author.

## Ethics statement

The studies involving human participants were reviewed and approved by Medical Ethical Committee of the University Hospitals and University of Leuven. The patients/participants provided their written informed consent to participate in this study.

## Author contributions

All authors listed have made a substantial, direct, and intellectual contribution to the work and approved it for publication.

## Funding

This work was partly funded by a research grant from Flanders Innovation & Entrepreneurship through the VLAIO research grant HBC.20192373, partly by a Wellcome Trust Collaborative Award in Science RG91976 to Robert P. Carlyon, John C. Middlebrooks, and JW, and partly by an SB Ph.D. grant 1S34121N from the Research Foundation Flanders (FWO) awarded to WD.

## Conflict of interest

The authors declare that the research was conducted in the absence of any commercial or financial relationships that could be construed as a potential conflict of interest.

## Publisher's note

All claims expressed in this article are solely those of the authors and do not necessarily represent those of their affiliated organizations, or those of the publisher, the editors and the reviewers. Any product that may be evaluated in this article, or claim that may be made by its manufacturer, is not guaranteed or endorsed by the publisher.
